# Peroxisome Proliferator-Activated Receptor-*γ* Prevents Cholesterol Gallstone Formation in C57bl Mice by Regulating Bile Acid Synthesis and Enterohepatic Circulation

**DOI:** 10.1155/2018/7475626

**Published:** 2018-07-12

**Authors:** Gang Wang, Tao Han, ShiJia Wang, Min Chen, Yueming Sun, Zan Fu

**Affiliations:** ^1^Department of Colorectal Surgery, The First Affiliated Hospital of NJMU, Nanjing 210029, China; ^2^Intensive Care Unit, The First Affiliated Hospital of NJMU, Nanjing 210029, China

## Abstract

To investigate the role of the peroxisome proliferator-activated receptor-*γ* (PPAR*γ*) in the progression of cholesterol gallstone disease (CGD), C57bl/6J mice were randomized to the following groups (n=7/group): L (lithogenic diet, LGD), LM (LGD+pioglitazone), CM (chow diet+pioglitazone), and NC (normal control, chow diet). Gallbladder stones were observed by microscopy. Histological gallbladder changes were assessed. Bile acids (BA) and cholesterol were measured in the serum, bile, and feces. Proteins and mRNA expression of genes involved in BA metabolism and enterohepatic circulation were assessed by western blotting and real-time RT-PCR. PPAR*γ* activation was performed in LO2 cell by lentivirus transfection and in Caco2 cell by PPAR*γ* agonist treatment. Downregulation of farnesoid X receptor (FXR) by small interference RNA (siRNA) was performed in L02 cells and Caco2 cells, respectively. Results showed that pharmacological activation of PPAR*γ* by pioglitazone prevents cholesterol gallstone formation by increasing biliary BA synthesis and enterohepatic circulation. Activated PPAR*γ* induced the expression of genes involved in enterohepatic circulation and bile acid synthesis (like PCG1*α*, BSEP, MRP2, MRP3, MRP4, NTCP, CYP7A1, CYP27A1, ASBT, OST*α*, and OST*β*). Downregulation of FXR repressed expression of partial genes involved in BA enterohepatic circulation. These findings suggest a new function of PPAR*γ* in preventing CGD by handling BA synthesis and transport through a FXR dependent or independent pathway.

## 1. Introduction

Cholesterol gallstone disease (CGD) is a multifactorial disease caused by the interaction of several poorly defined environmental and genetic factors [1]. As previously established, five conditions promote the formation of cholesterol crystals: (1) biliary cholesterol supersaturation due to hypersecretion of cholesterol by the liver [2]; (2) enhanced intestinal cholesterol absorption [2]; (3) relative reduction of BA and phospholipid content in the bile, with resultant decrease in bile hydrophilicity [2]; (4) biliary stasis due to impaired gallbladder motility, accompanied by gallbladder inflammation [3]; and (5) genetic defects [1, 4]. Cholesterol crystals precipitate when the balance among cholesterol, bile acids (BA), and bile phospholipids is disrupted [5]. These crystals keep aggregating and ultimately form pathologic gallstones. Cholesterol is only slightly soluble in aqueous media but is made soluble in bile through mixed micelles composed of bile salts and phospholipids [3]. Since enterohepatic circulation (EHC) plays a fundamental role in the regulation of the synthesis and transport of BA [6], a beneficial regulation of EHC could probably prevent CGD formation.

BA are amphipathic molecules which take great parts in intestinal nutrient absorption and biliary secretion of lipids, toxic metabolites, and xenobiotics. Bile acid pool size is maintained by two major mechanisms in healthy subjects, enterohepatic circulation, and de novo synthesis of bile acids. This latter mechanism compensates for the daily fecal loss of bile acids, whereas the majority of the pool is conserved by the former mechanism [6]. The cytochrome P450 enzymes (CYP) CYP7A1 [7], CYP8B1, and CYP27A1 [8] catalyze the biosynthetic process of cholesterol into BA. Both newly synthesized and reabsorbed bile acids are secreted into bile duct through membrane by highly specialized canalicular transporters [9]. The bile salt export pump (BSEP), also known as ABCB11, is the major canalicular BA transporter [10]. Cholesterol gallstone formation is associated with downregulation of BSEP expression on the canalicular membrane of hepatocytes [11]. Multidrug resistance protein 2 (Mrp2) is another transporter that facilitated BA transportation [12]. After a postprandial stimulus, gallbladder bile is secreted into duodenum for the promotion of the absorption of dietary lipids and lipid-soluble vitamins. At the distal ileum, the majority of the BA (95%) are efficiently reabsorbed via the apical sodium-dependent BA transporter (ASBT) and organic solute transporter *α*/*β* (Ost*α*/*β*) and then transported back to the liver through the hepatic uptake transporter sodium- (Na-) taurocholate cotransport protein (Ntcp) [13, 14]. Mrp3 and Mrp4 together with Ost*α*/*β* play roles in the sinusoid export of conjugated bile salts and promote their renal secretion [15, 16]. The farnesoid X receptor (FXR) [17, 18] plays an important role in regulating BSEP, multidrug resistance protein-2 (MRP2), ASBT, and Ost*α*/*β*, which are all involved in the enterohepatic circulation of BA [19].

The peroxisome proliferator-activated receptor-*γ* (PPAR*γ*), also called nuclear receptor 1C3 (NR1C3), is a member of the nuclear receptor superfamily of ligand-inducible transcription factors [20]. By binding to PPAR-responsive regulatory elements as obligate heterodimers with Retinoid X receptor, the PPARs control the expression of networks of genes involved in adipogenesis, lipid metabolism, inflammation, and metabolic homeostasis [21]. FXR interacts with PPAR*γ* and plays a part in adipocyte differentiation and lipid metabolism [22]. Pioglitazone, a PPAR*γ* agonist, is an oral antidiabetic agent that decreases blood glucose and lipids by improving insulin sensitivity [23]. In one of our previous studies, we found that PPAR*γ* was downregulated in LGD fed mice. While treating mice with pioglitazone could effectively prevent cholesterol gallstone formation [24], the mechanism remains unclear. We hypothesized that PPAR*γ* may act as a key regulator in preventing lithogenesis.

## 2. Materials and Methods

### 2.1. Animals, Lithogenic Diet, and Drug Treatments

Twenty-eight 6-week-old male C57bl/6J mice (Comparative Medicine Center of Yangzhou University) were randomly divided into four groups (n=7/group): group L (LGD), group LM (LGD+pioglitazone), group CM (chow diet+pioglitazone), and group NC (normal control, chow diet).

Mice in groups L and LM were fed a LGD containing 15% fat, 1.5% cholesterol, 0.5% cholic acid, and 18% casein, besides essential minerals and vitamins (Shanghai Pu Lu Teng Biological Technology Co., Ltd., China) [25]. The mice in groups CM and NC were fed standard chow diet. Pioglitazone (Pfizer Pharmaceuticals Limited., Jiangsu, China) (8 mg/kg) was used as an agonist of PPAR*γ* [26]. Intragastric administration of pioglitazone was performed once per day in the LM and CM groups 3 days prior to LGD and continued for 10 weeks. Distilled water was used as placebo in the NC and L groups, as negative control. All animals were housed in a temperature-controlled room under a 12-hour light/dark cycle with free access to water. The animal experiments were approved by the Ethics Committee of the First Affiliated Hospital of Nanjing Medical University, China (No. 2014-SRFA-066). The experimental procedures were carried out in accordance with the National Institutes of Health Guidelines for the Care and Use of Laboratory Animals. All animals were handled according to the guidelines of the First Affiliated Hospital of Nanjing Medical University Animal Research Committee.

### 2.2. Sample Collection

Feces were sampled for 3 days before sacrifice. Bile, liver tissues, serum, and intestinal tissues were collected immediately after sacrifice.

### 2.3. Microscopic Examination of Cholesterol Crystals

After intraperitoneal anesthesia with chloral hydrate, cholecystectomy was performed after 10 weeks of LGD. After a 10-h fast, intact gallbladders were excised after ligation of the common bile duct. The gallbladder bile was collected and stored at -20°C. Bile (1 *μ*L) was evenly spread on glass slides and examined under an Axio Imager A1 microscope (Carl Zeiss GmbH, Oberkochen, Germany). The total number of visible crystals was determined under polarizing light mode.

### 2.4. Fecal BA Extraction

Feces were collected from individually housed mice over 48 h. Fecal BA were extracted with 75% ethanol at 50°C for 2 h and centrifuged at 1500 *g* for 10 minutes. The supernatant phase was collected and assessed using an ELISA kit (Shenzhen Hui Jia Biological Co., Ltd., China).

### 2.5. Histological Examination

Gallbladder tissues were soaked in 4% formaldehyde, embedded in paraffin, sectioned at 5 *μ*m, and stained with hematoxylin-eosin. The tissue sections were examined with an Axio Imager A2 microscope (Carl Zeiss GmbH, Oberkochen, Germany).

### 2.6. Serum, Biliary, and Fecal Biochemical Parameters

A Hitachi 7100 fully automatic biochemical analyzer was used for measurement of cholesterol and BA in the serum, bile, and fecal lipid samples. Plasma levels of alanine aminotransferase (ALT), aspartate aminotransferase (AST), high-density lipoprotein cholesterol (HDL-C), low-density lipoprotein cholesterol (LDL-C), and triglycerides (TG) were measured. Bile phospholipids were measured with an ELISA kit (Biohjsw, Xiamen, China), according to the manufacturer's instructions. The cholesterol saturation index (CSI) was calculated according to Carey's critical tables [27].

### 2.7. Cell Culture

The human hepatocytes cell line LO2 was provided by the Liver Transplantation Center of the Jiangsu Province Hospital. Caco2 cell was provided by the colorectal surgery department of the Jiangsu Province Hospital. Cells were grown in Dulbecco's modified Eagle's medium (DMEM; GIBCO, Invitrogen Inc., Carlsbad, CA, USA) supplemented with 10% fetal bovine serum (FBS; GIBCO, Invitrogen Inc., Carlsbad, CA, USA) and antibiotics in a 5% CO_2_ atmosphere at 37°C. Chemical treatments: The PPAR*γ* agonist pioglitazone was dissolved manually in DMEM. Caco2 cell cultures were exposed to 10*μ*M and lasted for 24h. At least three independent experiments were performed.

### 2.8. Lentivirus Infection

Recombinant lentiviruses overexpressing PPAR*γ* (LV-PPAR*γ*) and negative control vector (LV-NC) were obtained from Genechem (Shanghai Genechem Co, Ltd., Shanghai, China). Two PPAR*γ* targeting primer sequences were designed: P1, 5'-CCA ACT TTG TGC CAA CCG GTC GCC ACC ATG ACC ATG GTT GAC ACA GAG ATG-3', and P2, 5'-AAT GCC AAC TCT GAG CTT GTA CAA GTC CTT GTA GAT CTC CTG-3'. Lentivirus transduction was performed according to the manufacturer's protocol. Transduced cells were subjected to puromycin selection for one week. Successful overexpression of PPAR*γ* in stably transduced cells was confirmed by real-time quantitative polymerase chain reaction (qRT-PCR). The stable cell lines were designated as LO2pre and LO2NC, respectively.

### 2.9. siRNA Transfection

The siRNAs (Genepharma Co., Ltd., Suzhou, China) targeting FXR were constructed according to the human FXR sequences (FXR: BC035654). We designed multiple siRNAs in order to avoid off-target effect (Supplementary Table 1). Finally, the siRNAs with the highest transfection efficiency (FXR-homo-1282) were selected for the subsequent experiments. Scramble siRNA was provided by Genepharma Co., Ltd. Transfections were performed with Lipofectamine 2000 reagent (Invitrogen Inc., Carlsbad, CA, USA), according to the manufacturer's protocol. Cells were plated on 6-well plates at a confluence of 70-90% per well. Transfection of each well was done using 10 *μ*M siRNA, together with 2 mL complete culture medium without antibiotics. The successfully transfected cells were collected for real-time RT-PCR examination.

### 2.10. Real-Time RT-PCR

Total RNAs from tissues and cells were extracted with TRIzol (Invitrogen Inc., Carlsbad, CA, USA). RNA purity was determined using absorbance at 260 and 280 nm (A260/280) using a Thermo Scientific NanoDrop 2000 spectrophotometer. Total RNA was reverse transcribed into cDNA using a PrimeScript Master Mix kit (Takara Biotechnology, Otsu, Japan). Real-time PCR using a FastStart Universal SYBR Green Master (Rox) (Roche Diagnostics, Basel, Switzerland) was performed using a Roche LightCycler 96 system. Primer sequences are available in Supplementary Table 2. Glyceraldehyde-phosphate dehydrogenase (GAPDH) was used for normalizing data, and the real-time PCR amplification efficiency of target genes was considered when using LightCycler® 96 Application Software version 1.1 for data analysis. 2^−ΔΔCt^ was calculated to represent the relative mRNA expression of target gene.

### 2.11. Statistical Analyses

All statistical analyses were conducted using SPSS 19.0 (IBM, Armonk, NY, USA). Data were represented as mean ± standard error of the mean (SEM). Statistical significance was evaluated by two-tailed unpaired* t*-tests or one-way analysis of variance (ANOVA). Two-sided P values <0.05 were considered statistically significant.

## 3. Results

### 3.1. Pioglitazone Attenuates Cholesterol Crystallization and Gallbladder Inflammation in Mice Fed on LGD

**Figure 1:**
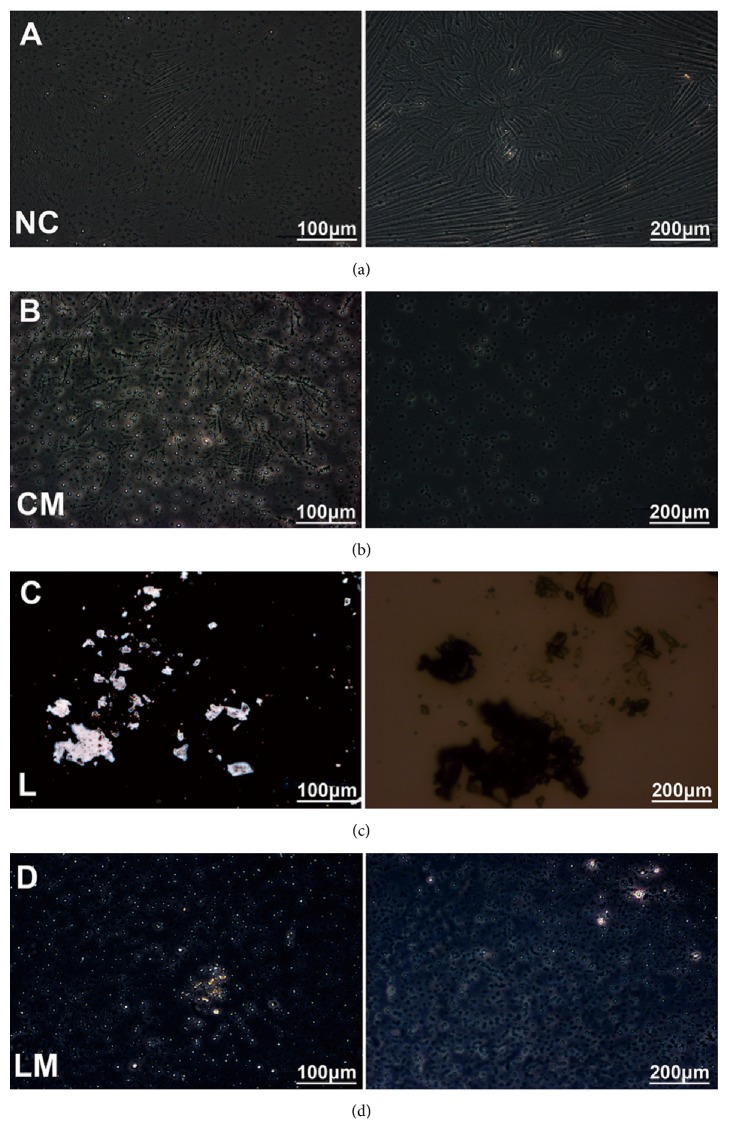
**Pioglitazone prevented cholesterol gallstone formation in mice fed on a lithogenic diet (LGD).** Mice were randomly divided into four groups: group L (LGD), group LM (LGD+pioglitazone), group CM (chow diet+pioglitazone), and group NC (normal control, chow diet). Cholesterol crystals were examined under polarizing light microscope in groups NC (a), CM (b), L (c), and LM (d).

We did not observe any cholesterol crystals in mice fed with standard chow diet, with or without pioglitazone treatment (groups NC and CM) (Figures 1(a) and 1(b)). LGD significantly induced the formation of micro gallstones and solid cholesterol monohydrate crystals in group L (Figure 1(c)). Pioglitazone treatment significantly slowed down the formation of cholesterol crystals, and no micro gallstones were found in the LM group despite LGD treatment (Figure 1(d)). The gallstone formation rate in group L was 100% (7/7), whereas in the LM, NC, and CM groups it was 14.3% (1/7), 0% (0/7), and 0% (0/7), respectively.

**Figure 2:**
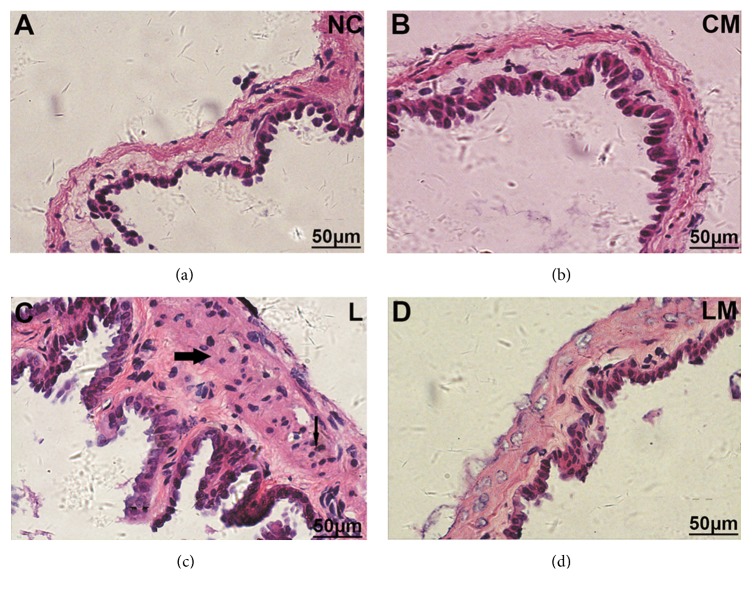
**Pioglitazone alleviated gallbladder inflammation in mice fed on LGD. **Histological examination of gallbladder was determined by hematoxylin-eosin staining in groups NC (a), CM (b), L (c), and LM (d). Slim arrows indicate stromal granulocyte infiltration. Thick arrows indicate the thickness of the gallbladder wall.

Gallbladder inflammation is indicated by thickened gallbladder wall, infiltrated inflammatory cells in the stromal layer, and submucosal vasodilatation, and these are another hallmark of CGD [28]. None of these signs were detected in the NC and CM groups (Figures 2(a) and 2(b)), but all these signs were seen in the L group (Figure 2(c)). Few inflammatory signs were observed in the LM group (Figure 2(d)).

### 3.2. Pioglitazone Altered the Biochemical Composition of the Gallbladder Bile and Improved Biliary CSI in Mice Fed on LGD

**Figure 3:**
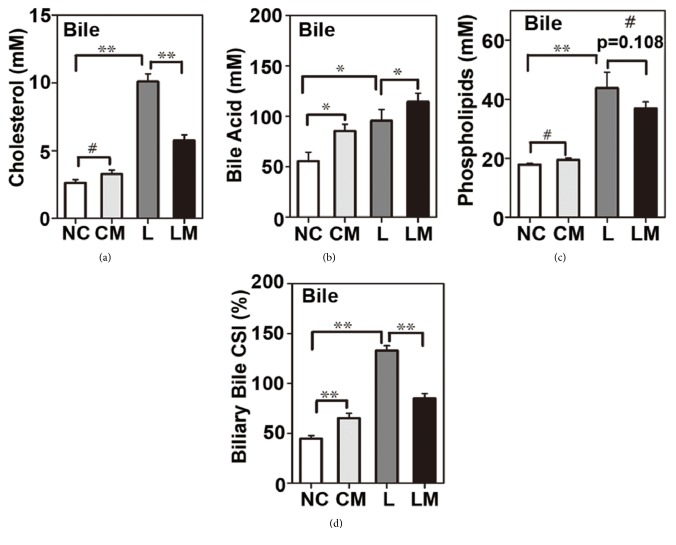
**Pioglitazone altered gallbladder bile profiles, leading to a decreased biliary cholesterol saturation index (CSI) in mice fed on LGD.** (a) Biliary cholesterol concentration, (b) biliary bile acid (BA) concentration, (c) biliary phospholipid concentration, and (d) biliary CSIs calculated according to parameters in panels (a–c). Results are presented as mean ± standard error of the mean (SEM) (n=7/group). *∗P*<0.05, *∗∗P*<0.01, and #*P*>0.05.

Standard chow diet had few effects on the metabolic parameters of the NC and CM groups. In the LGD treated groups, metabolic parameters like body weight, body fat mass (%), liver weight (%), total cholesterol, and triglycerides were increased, whereas these metabolic parameters were improved by pioglitazone in the LM group compared to their L group counterparts, except the percentage of liver weight (Table 1). Nevertheless, in all groups, liver weight ratio was a little below 4%, which is considered the normal liver weight ratio in mice [29]. LGD significantly increased biliary cholesterol content in the L group, but pioglitazone restored this increase in the LM group (Figure 3(a)). Pioglitazone improved biliary BA content in the LM and CM groups compared to the L and NC groups, respectively (Figure 3(b)). LGD treatment induced the content of phospholipids, but there was no significant difference between the L and LM groups and a similar trend was observed in the NC and CM groups (Figure 3(c)). Therefore, higher content of BA together with lower content of cholesterol in bile in the LM group led to decreased bile CSI compared to the L group (Figure 3(d)). However, the CM group displayed a slightly higher CSI than the NC group.

### 3.3. Pioglitazone Altered BA Canalicular Transport and Reabsorption Responsive Genes Expression in Liver and Intestine

**Figure 4:**
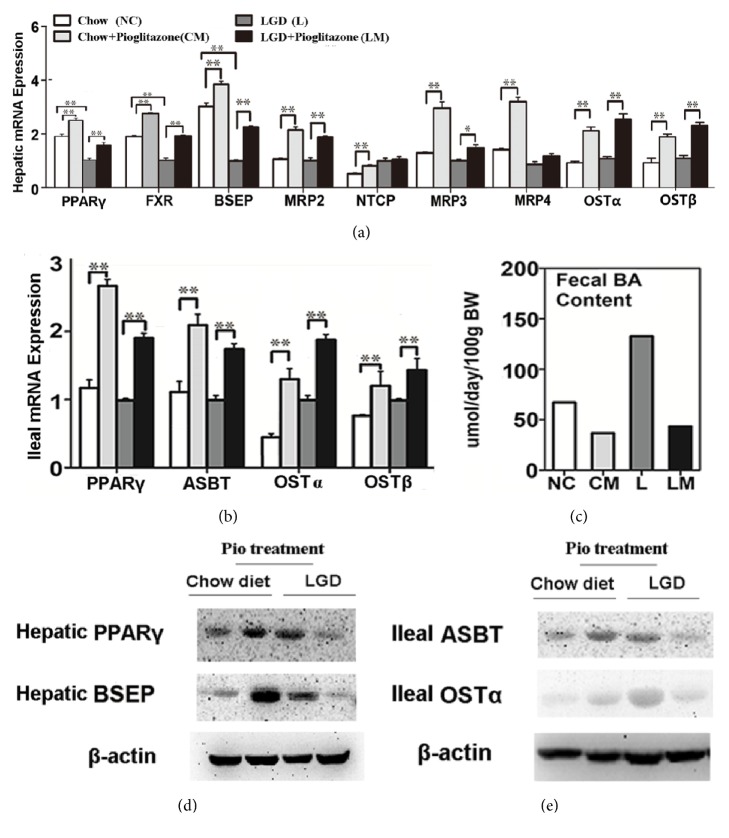
**Pioglitazone altered BA canalicular transport and reabsorption responsive genes expression in liver and intestine.** (a) mRNA expressions of hepatic PPAR*γ*, bile salt export pump (BSEP), multiple drug resistance protein- (MRP-) 2 and Na+/taurocholate cotransporting polypeptide (NTCP), MRP3 and MPR4 (BA transporters excreting BA into sinusoid), and organic solute transporter *α*/*β* (Ost*α*/*β*), as determined by real-time RT-PCR. (b) mRNA expressions of ileal PPAR*γ*, apical sodium/bile acid cotransporter (ASBT), and organic solute transporter *α*/*β* (Ost*α*/*β*) (involved in BA reabsorption), as determined by real-time RT-PCR. (c) Fecal BA content was detected and measured with a fully automatic analyzer. (d) Western blotting results of hepatic PPAR*γ* and BSEP. (e) Western bolting results of ileal ASBT and OST*α* data are shown as mean ± SEM (n=7/group). *∗P*<0.05; *∗∗P*<0.01.

Consistent with our previous study [24], LGD treatment suppressed PPAR*γ* expression, while pioglitazone treatment restored the expression of PPAR*γ* (Figures 4(a) and 4(d)). LGD suppressed the expression of BSEP, while pioglitazone induced its expression in both the LM and CM groups (Figures 4(a) and 4(d)). Other BA transporters like MRP2, MRP3, MRP4, and Ost*α*/*β* were also induced in the pioglitazone-treated groups (Figure 4(a)). Western blotting results of PPAR*γ* and BSEP revealed higher expressions in pioglitazone-treated groups in comparison to their own counterparts, respectively (Figure 4(c)).

Given that enhanced intestinal BA reabsorption plays an important role in the pathological process of gallstone formation [30], we investigated the expression of BA transporters in ileum tissue, including the majority transporter ASBT and efflux transporters OST*α*/*β*. LGD treatment slightly induced the expression of OST*α*, OST*β*, and ASBT, but pioglitazone significantly increased the expression of OST*α*/*β* (Figure 3(b)). Pioglitazone also induced the expression of ASBT (Figure 3(b)), and the fecal BA concentration was decreased following the pioglitazone treatment in the LM and CM groups (Figure 4(c)). Increased protein expression of ASBT and OST*α* was detected by western blot assay in drug-treated mice (Figure 4(e)).

### 3.4. Pioglitazone Enhanced BA Synthesis in Mice Fed on LGD

**Figure 5:**
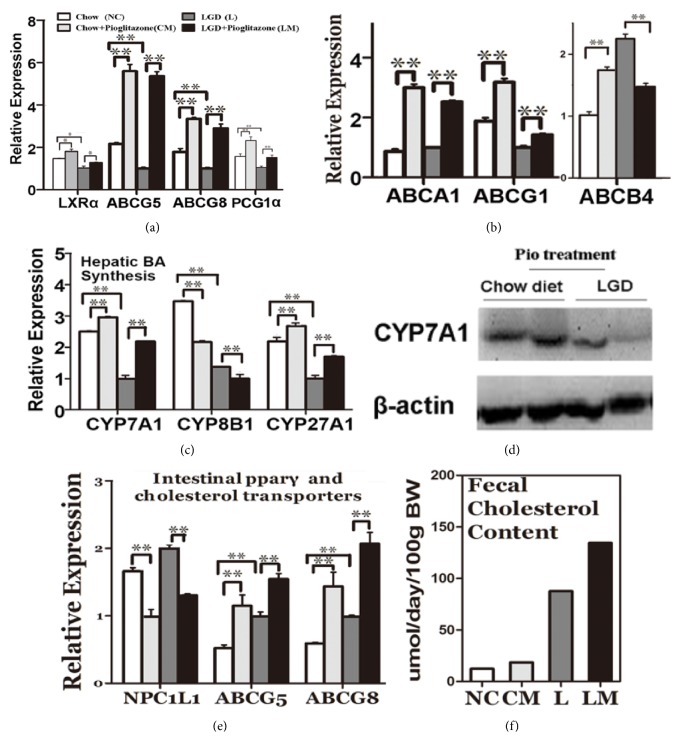
**Effects of activation of PPAR** **γ** **on expressions of genes involved in BA synthesis and cholesterol transport in liver tissues of mice fed on LGD**. (a, b) mRNA expressions of hepatic liver X receptor *α*, peroxisome proliferator-activated receptor-*γ* coactivator *α* (PGC-1*α*), and hepatic cholesterol and phospholipid transporters (ATP-binding cassette transporter G5 (ABCG5), ABCG8, ABCA1, ABCG1, ABCB4). (c) Key enzymes involved in BA synthesis (cytochrome P450 enzyme 7A1 (CYP7A1), CYP8B1, CYP27A1), as determined by real-time RT-PCR. (d) Western blotting results of hepatic CYP7A1. (e) mRNA expressions of intestinal cholesterol transporters (Niemann-Pick C 1-like 1 (NPC1L1), ABCG5, and ABCG8). (f) Fecal cholesterol content was detected and measured with a fully automatic analyzer. Data are presented as the means ± SEM (n=7/group). *∗P*<0.05; *∗∗P*<0.01.

LXR*α* is one of the key regulators in cholesterol and BA metabolism, which were suppressed by LGD but induced by pioglitazone in the drug-treated groups (Figure 5(a)). Cholesterol efflux is facilitated by Abcg5 and Abcg8 [31]. This heterodimer was also suppressed by LGD, while pioglitazone significantly induced their expression (Figure 5(a)). Peroxisome proliferator-activated receptor-*γ* coactivator *α* (PGC-1*α*) is known as a key regulator in many metabolic processes like adaptive thermogenesis, mitochondrial biogenesis, and hepatic gluconeogenesis, among others. The expression of PGC-1*α* could also be suppressed by LGD but induced by pioglitazone in the liver (Figure 5(a)). Abcb4 is responsible for canalicular efflux of phospholipids, whereas Abca1 and Abcg1 are basolateral transporters which participate in cholesterol reverse transportation [32, 33]. Expression of Abca1 and Abcg1 was induced by pioglitazone treatment. Meanwhile Abcb4 displayed paradoxical results (Figure 5(b)).

BA de novo synthesis is catalyzed by multiple CYP enzymes. We found the mRNA expression of Cyp7a1 and Cyp27a1, the two key enzymes in bile acid synthesis, was markedly suppressed in lithogenic diet-fed mice, while pioglitazone restored their expression. CYP8b1 was detected with decreased expression in pioglitazone-treated mice (Figure 5(c)). The induced expression of Cyp7a1 protein expression was confirmed by western blot analysis (Figure 5(d)).

### 3.5. Pioglitazone Reduced Intestinal Cholesterol Absorption in Mice Fed on LGD

Fecal cholesterol was detected and it was found that pioglitazone significantly increased the fecal cholesterol output in the LM group compared to the L group (Figure 5(f)). Therefore, we investigated the expression of cholesterol transporters in intestinal tissues, including the efflux transporters ABCG5/ABCG8 and the cholesterol absorption transporter Niemann-Pick C1-like 1 (NPC1L1) protein. LGD treatment slightly induced the expression of ABCG5/ABCG8 and NPC1L1, but pioglitazone significantly elevated the expression of ABCG5/ABCG8. Conversely, pioglitazone reduced the expression of NPC1L1 (Figure 5(e)).

### 3.6. PPAR*γ* Regulates Genes Involved in EHC in LO2 and Caco2 Cells by FXR Dependent Mechanisms

**Figure 6:**
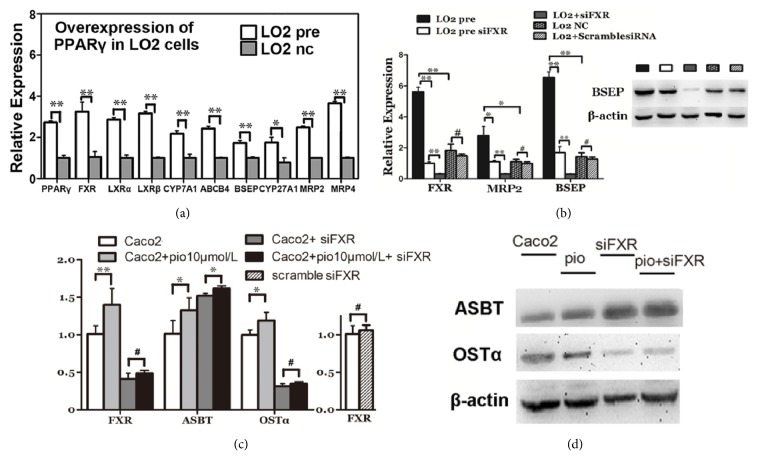
**PPAR*γ* regulates genes involved in EHC in LO2 and Caco2 cell lines by FXR dependent mechanisms.** LO2 cells were stably transfected with PPAR*γ* overexpression (L02pre) or negative control vector (L02NC). (a) mRNA expression of PPAR*γ*, LXR*α*, LXR*β*, FXR, BSEP, ABCB4, CYP7A1, CYP27A1, MRP2, and MRP4 in PPAR*γ*-overexpressed L02 cells, as determined by real-time RT-PCR. L02pre were further transfected with FXR siRNA. (b) mRNA expression of FXR, BSEP, and MRP2 was determined by real-time RT-PCR. GAPDH was used for data normalizing. Western blotting was performed on BSEP. (c) 10*μ*M pioglitazone was used for activation of PPAR*γ*, and siRNA targeting FXR was used for downregulating FXR. mRNA expression of FXR, OST*α*, and ASBT was determined by real-time RT-PCR. (d) Western blotting was performed on ASBT and OST*α* in Caco2 cell treated as previously established. Data are presented as the means ± SEM of three independent experiments performed in duplicate. *∗P*<0.05, *∗∗P*<0.01, and #*P*>0.05.

After overexpression of PPAR*γ*, FXR, LXR*α*/*β*, and genes involved in hepatic BA transportation (such as BSEP, MRP2, and MRP4) were significantly induced (Figure 6(a)). Genes involved in BA synthesis like CYP7A1 and CYP27A1 were also detected with induced expression (Figure 6(a)). Knock-down of FXR in both Lo2 pre and Lo2 cells displayed interesting results. The expression of BSEP and MRP2 decreased when FXR was knocked down, and the induced expression of BSEP and MRP2 in Lo2-pre cells was restored. Western blotting results revealed decreased protein expression of BSEP following knocking down of FXR (Figure 6(b)).

Induced expression of ASBT and OST*α* was detected in pioglitazone-treated Caco2 cells in comparison to no drug-treated counterparts. After knocking down of FXR, the induced expression of OST*α* was restored. The expression of ASBT was increased due to repression of FXR, and this was consistent with previous studies that FXR inhibits the expression of ASBT. Western blotting results displayed the same alteration of ASBT and OST*α* expression on protein levels (Figure 6(d)).

## 4. Discussion

**Figure 7:**
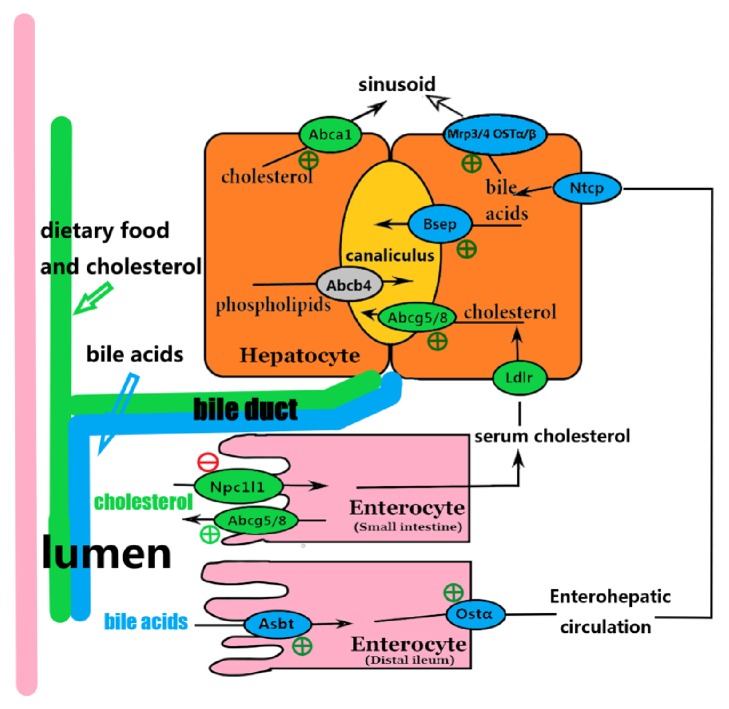
**Summary of the PPAR*γ* effects on cholesterol and bile salt transporter genes expression.** Transporters in blue ovals are upregulated by PPAR*γ* activation in the presence of lithogenic diet treatment, except Ntcp. Transporters in green ovals are also upregulated by PPAR*γ* activation in the presence of lithogenic diet treatment, except Ldlr and Npc1l1. Npc1l1 is downregulated by PPAR*γ* activation. The net effect of PPAR*γ* activation is increased biliary concentration of bile salts and increased fecal cholesterol content as well as decreased content of fecal bile salts. Abc: adenosine triphosphate binding cassette; ASBT: sodium-dependent bile acid transporter; BSEP: bile salt export pump; Ldlr: low-density lipoprotein receptor; Mrp: multidrug resistance protein; Npc1l1: Niemann-Pick C1 like 1; Ntcp: Na-taurocholate cotransport proteins; Ost: organic solute transporter.

In this report, lithogenic mice models were made; human hepatocyte L02 and human colon cell Caco2 were cultured in order to investigate the role of PPAR***γ*** in preventing lithogenesis. Pharmacological activation of PPAR***γ*** by pioglitazone inhibited lithogenesis in C57bl mice, which was associated with an increased biliary concentration of bile acids and enhanced enterohepatic circulation. And this regulatory function of PPAR***γ*** on BA enterohepatic circulation was through a FXR dependent pathway.  Figure 7 presents a summary of the genes involved.

In the present study, the LGD was high in fat and cholesterol, and it was fed to mice for 10 weeks. In these conditions, the LGD can induce weight gain and obesity, as previously observed [24, 26, 34], while pioglitazone administration improved body weight, adipose mass, serum cholesterol, and serum triglyceride. Decreased LDL and increased HDL levels were also detected in pioglitazone-treated mice, respectively (Table 1). This might be due to the positive function of PPAR***γ*** in improving adipose differentiation and insulin sensitivity [35]. Interestingly, serum content of BA in LM group mice was much higher than in L group. This might indicate increased hepatic sinusoidal efflux of BA. We attributed this to the induced expression of OST*α*/*β*. These two proteins act as bile acid efflux transporters and transport bile acids into the sinusoidal blood to prevent bile acid accumulation in the hepatocytes. MRP3 and MRP4 also transport bile acids from the hepatocytes to the serum like OSTs, but at lower rates [15, 16]. Serum ALT and AST were increased in the LM group mice, which could be due to a slight toxic effect of pioglitazone.

Higher gallbladder biliary BA content in pioglitazone-treated mice was detected, and we reason this to enhanced hepatic BA transport. BSEP is the major hepatic BA canalicular efflux transporter. MRP2 mediates the transport of a wide range of organic substrates and also shows substrate specificity for divalent bile acids. Both BSEP and MRP2 are FXR target genes [36, 37]. We showed that mRNA expression of FXR, BSEP, and MRP2 was dramatically induced following overexpression of PPAR*γ* in L02 cells. Nevertheless, the expressions of BSEP and MRP2 were restored after FXR-specific knock-down. Our results indicated that the mechanism by which PPAR*γ* regulates the expression of BSEP and MRP2 was FXR dependent. Interestingly, induced expression of PGC-1*α* was detected in pioglitazone-treated mice. FXR could be induced through the coactivation of PPAR*γ* and HNF4. Thus, the expression of BSEP and MRP2 might be regulated through the PPAR*γ*-PGC-1*α*-FXR pathway [38]. Significantly lower fecal BA content in pioglitazone administrated mice indicated an enhanced intestinal BA reabsorption which is another important part of BA enterohepatic circulation. We interestingly found that ileal FXR, ASBT, and OST*α* could be induced by pioglitazone treatment in Caco2 cells. While increased ASBT but decreased OST*α* expressions were detected following downregulation of FXR, respectively. Results showed that PPAR*γ* could regulate OST*α* by FXR dependent mechanism but not ASBT. Activated FXR could inhibit the expression of ASBT in mice which was previously reported [39]. Though PPAR*γ* could induce the expression of ASBT, the mechanism needs further study.

As to the mechanism for the increased bile acid pool size in the pioglitazone-treated mice, one interesting finding is the induced expression of Cyp7a1. It is known that the expression of Cyp7a1 can be regulated by several negative feedback loops. There is a feedback repression of Cyp7a1 when feeding mice with cholic acid which was contained in LGD. FXR is also known to suppress BA synthesis via the FXR-SHP-LRH-1 pathway [40]. Our results displayed induced expression of CYP7A1 and CYP27A1 following activation of PPAR*γ* in both mice and L02 cells despite the induced expression of FXR. Interestingly, genes involved in BA synthesis (such as CYP7A1 and CYP27A1) are positively regulated by LXR*α* [41, 42]. Liver X receptors were initially characterized as sterol sensors which participated in cholesterol and lipid homeostasis [43]. In rodents, LXR*α* promotes BA synthesis by inducing the expression of CYP7A1 and can also promote BA detoxification and alleviate cholestasis [44, 45]. Induced expression of LXR*α* by PPAR*γ* was detected. The regulation of BA synthesis is complex, but our results nevertheless indicated a synergistical action of LXR*α* and FXR. LXR*α* might play an effective role in improving BA synthesis. Since we used a diet rich in CA and cholesterol, the diet could also be prone to generate ligands for LXR via CYP27 [46, 47]. Nevertheless, the present study was not designed to examine this point and it will have to be explored in future studies.

Due to the induced expression of LXR*α*, expressions of ABCG5/ABCG8 were induced. Interestingly, the biliary levels of cholesterol in the LM and CM groups were not greatly affected. Induced expression of ABCA1 may lead to enhanced cholesterol efflux [33], but we did not observe significantly higher serum cholesterol levels in pioglitazone-treated mice. We therefore hypothesized that intestinal cholesterol absorption was inhibited. Cholesterol intake is selectively mediated by NPC1L1. It is one of the most important proteins involved in the regulation of cholesterol absorption by the intestine [48]. Previous studies demonstrated that inhibition of intestinal NPC1L1 prevents gallstone formation; in addition, ezetimibe, a selective NPC1L1 inhibitor, prevents gallstone formation in mice, indicating that NPC1L1 is a valid therapeutic target against CGD [26]. ABCG5/G8, heterodimeric member of the ABC transporter superfamily, are also expressed in the intestine. Interestingly, their functions are the opposite to that of NPC1L1 and they act as a “pump” to move excess cholesterol, together with nonesterified cholesterol and phytosterols, back into the intestinal lumen. Therefore, the activation of ABCG5/G8 decreases the body cholesterol levels [49]. Reduced expression of NPC1L1 and induced expression of ABCG5/G8 were detected in pioglitazone-treated mice. These results might account for the increased fecal cholesterol output in pioglitazone-treated mice and indicated inhibited intestinal cholesterol absorption.

The present study is not without limitations. First, the effects of the LGD on obesity and lipid parameters shown in this study are not completely consistent with the known effects of atherogenic diets in mice. This could be due to differences in the diets themselves among studies, as well as the genetic background of the mice and the sample size. Secondly, pioglitazone was used for pharmacological activation of PPAR*γ* but was found to be associated with bladder tumors and withdrawn by a few countries. Thirdly, there is a possibility that the prevention of CGD was a secondary result of generally improved lipid homeostasis by pioglitazone. These experiments could better be performed in tissue-specific PPAR*γ* KO mice. And these issues will be examined in future studies.

In conclusion, the present study suggests a new function of PPAR*γ* in preventing lithogenesis. Genes involved in BA synthesis and enterohepatic circulation could be widely induced by PPAR*γ*. The regulation of BSEP, MRP2, and OST*α* is in a FXR dependent pathway. Future investigation of PPAR*γ* may help clarify the pathogenesis of metabolic syndromes in humans and provide new methods for the prevention or treatment of CGD.

## Figures and Tables

**Table 1:** Pioglitazone alleviates diet-induced obesity and biochemical parameters in mice fed on LGD.

**Variables**	**Group NC**	**Group CM**	**Group L**	**Group LM**
**Body weight (g)**	29.8 ± 1.2	27.4 ± 1.3*∗*	33.0 ± 1.8#*∗*	29.2 ± 2.0Δ
**Body adipose mass (g)**	0.69 ± 0.18	0.61 ± 0.14	1.92 ± 0.82#*∗*	0.93 ± 0.24Δ*∗*
**Liver weight (g)**	0.89 ± 0.11	0.86 ± 0.14	1.16 ± 0.22#*∗*	1.04 ± 0.18Δ*∗*
Liver** weight/ body weight (**%**)**	2.98 ± 0.20	3.11 ± 0.28	3.56 ± 0.38*∗*	3.51 ± 0.30*∗*
A**dipose mass/body weight (**%**)**	2.31 ± 0.39	2.20 ± 0.33	5.73 ± 1.70#*∗*	3.13 ± 0.48Δ*∗*
**Total cholesterol (mmol/L)**	2.88 ± 0.25	2.78 ± 0.18	4.82 ± 1.07#*∗*	4.04 ± 0.45#*∗*
**Triglycerides (mmol/L)**	0.60 ± 0.10	0.59 ± 0.20	1.14 ± 0.28*∗*	0.80 ± 0.08*∗*
**Blood bile acid (** ***μ*** **mol/L)**	8.83 ± 4.57	9.33 ± 5.92	7.47 ± 2.0	10.70 ± 5.85Δ
**HDL (mmol/L)**	2.17 ± 0.20	2.00 ± 0.11	2.84 ± 0.26#*∗*	3.15 ± 0.68#*∗*
**LDL (mmol/L)**	0.72 ± 0.38	0.67± 0.16	1.12 ± 0.26	0.86 ± 0.12
**ALT (U/L)**	26.5 ± 3.6	28.1 ± 1.1	28.6 ± 4.8	30.3 ± 3.6*∗*
**AST (U/L)**	124.5 ± 22.8	112.5 ± 14.7	134.3 ± 25.5*∗*	143.7 ± 37.6*∗*

Data are presented as mean ± standard error of mean (SEM) (n=7 for each group). *∗P*<0.05 vs. group NC; #*P*<0.05 vs. group CM; Δ*P*<0.05vs. group L. HDL, high-density lipoprotein; LDL: low-density lipoprotein; ALT: alanine aminotransferase; AST: aspartate aminotransferase. Group L, lithogenic diet (LGD) treatment group; group LM, LGD plus pioglitazone treatment group; group CM, chow diet plus pioglitazone treatment group; group NC, control group (neither LGD nor pioglitazone treatment).

## Data Availability

The datasets used and/or analyzed during the current study are available from the corresponding author on reasonable request.

## Supplementary Materials

Supplementary Materials The information of siRNAs targeting FXR will be found in Supplementary Table 1. The information of primer sequences used for real-time PCR will be found in Supplementary Table 2.
