# Supplementary material for: Peroxisome Proliferator-Activated Receptor-γ Prevents Cholesterol Gallstone Formation in C57bl Mice by Regulating Bile Acid Synthesis and Enterohepatic Circulation
Source: Biomed Res Int. 2018 Jul 12;2018:7475626. doi: 10.1155/2018/7475626 (PMC6076980; doi:10.1155/2018/7475626)
Supplement: Supplementary Materials — The information of siRNAs targeting FXR will be found in Supplementary Table 1. The information of primer sequences used for real-time PCR will be found in Supplementary Table 2. [file 7475626.f1.pdf]

Supplementary table 1. siRNAs targeting LXRα and FXR

siRNA	Primer (5'-3')
LXRa-homo-568	F: GCUUCCACUACAAUGUUCUTT
	R: AGAACAUUGUAGUGGAAGCTT
LXRa-homo-1045	F: GCAGGAGAUAGUUGACUUUTT
	R: AAAGUCAACUAUCUCCUGCTT
LXRa-homo-1176	F: GGAGUGAGAGUAUCACCUUTT
	R: AAGGUGAUACUCUCACUCCTT
FXR-homo-1070	F: CAGGCUUGUUAACUGAAAUTT
	R: AUUUCAGUUAACAAGCCUGTT
FXR-homo-1282	F: CCUCAGGAAAUAACAAAUATT
	R: UAUUUGUUAUUUCCUGAGGTT
FXR-homo-711	F: GUGGUACUCUCCUGGAAUATT
	R: UAUUCCAGGAGAGUACCACTT
Scramble	F: UUCUCCGAACGUGUCACGUTT
	R: ACGUGACACGUUCGGAGAATT

F: Forward; R: Reverse; LXRα: liver X receptor α; FXR: farnesoid X receptor

Supplementary table 2. Primer sequences for real-time PCR

Gene	Primer sequences (5'-3')
mPPARγ*	F: TCTCAGTGGAGACCGCCCAGG
mPPARγ*	R: GCTGCACGTGCTCTGTGACGAT
mCyp7a1	F: GTCCGGATATTCAAGGATGCA
mCyp7a1	R: AGCAACTAAACAACCTGCCAGTACTA
mABCG5	F: TGCCCATTCCTTTAAAAATCC
mABCG5	R: GATGAACTGGACCCCTTGG
mABCG8	F: GTAGCTGATGCCGATGACAA
mABCG8	R: GGGGCTGATGCAGATTCA
mABCA1	F: AATTCTCAAGTGCAAACACTTCTGG
mABCA1	R: GAGGCATATGCTTGCGGTACA
mABCG1	F: GTCTCAGCCTTCTAAAGTTCCTC
mABCG1	R: TCTCTCGAAGTGAATGAAATTTATCG
mLXR	F: TAGGGATAGGGTTGGAGTCAG
mLXR	R: AGTCAACAATCTCCTGCACGG
mBSEP	F: TGGAAAGGAATGGTGATGGG
mBSEP	R: CAGAAGGCCAGTGCATAACAGA
mLDLR	F: CTGTGGGCTCCATAGGCTATCT
mLDLR	R: GCGGTCCAGGGTCATCTTC
mSR-B1	F: GGCTGCTGTTTGCTGCG
mSR-B1	R: GCTGCTTGATGAGGGAGGG
mMrp2	F: TCCAGGACCAAGAGATTTGC
mMrp2	R: TCTGTGAGTGCAAGAGACAGGT
mMrp3	F: AGTGGCTGTGATAGTCTTGCTGATA
mMrp3	R: CCGTTCAGGATCTCGCTCAT
mMrp4	F: ACCTCTGCTCGCGCGTGTTCT
mMrp4	R: CCAGTACCGTTGAAGCTCCTCTCC
mAbcb4	F: CCGCTATGGCCGTGGGAATGTAA
mAbcb4	R: ACTCAGCTGCGCCCCTCTATCACC
mNpc1l1	F: AGTGCGGTGTTTGCTGGAGTGG
mNpc1l1	R: AGGAGGTTGAGGCGGAAGAAGAAA
mASBT	F: TGGGTTTCTTCCTGGCTAGACT
mASBT	R: TGTTCTGCATTCCAGTTTCCAA
mNTCP	F: GCATGATGCCACTCCTCTTATAC
mNTCP	R: TACATAGTGTGGCCTTTTGGACT
mFXR*	F: TGGGTACCAGGGAGAGACTG
mFXR*	R: GTGAGCGCGTTGTAGTGGTA
mCYP27A1	F: GACAACCTCCTTTGGGACTTAC
mCYP27A1	R: GTGGTCTCTTATTGGGTACTTGC
mOstα	F: TTGTGATCAACCGCATTTGT
mOstα	R: CTCCTCAAGCCTCCAGTGTC
mOstβ	F: ATCCTGGCAAACAGAAATCG

mOstβ	R: GGCCAAGTCTGGTTTCTCTG
mPCG1α	F: GAAAGGGCCAAACAGAGAGA
mPCG1α	R: GTAAATCACACGGCGCTCTT
mLXR α	F: TAGGGATAGGGTTGGAGTCAG
mLXR α	R: AGTTTCTTCAAGCGGATCTGT
mCYP8B1	F: GAATCTAACCAGGCCATGCT
mCYP8B1	R: AGGAGCTGGCACCTAGACT
mGADPH	F: GCTCGGCCGGCTGGAAGAACT
mGADPH	R: CCCTCGTTCTGCACGCGGAT
hPPARγ	F: GCTGTGCAGGAGATCACAGA
hPPARγ	R: GGGCTCCATAAAGTCACCAA
hLXRα	F: AAGCCCTGCATGCCTACGT
hLXRα	R: TGCAGACGCAGTGCAAACA
hLXRβ	F: TCGTGGACTTCGCTAAGCAA
hLXRβ	R: GCAGCATGATCTCGATAGTGGA
hFXR	F: TCGTGGACTTCGCTAAGCAA
hFXR	R: TCTCCTGGGTCGCCTGACT
hBSEP	F: ACATGCTTGCGAGGACCTTTA
hBSEP	R: GGAGGTTCGTGCACCAGGTA
hMRP2	F: GGCAGTGAAGAAGAAGACGATGA
hMRP2	R: ATTGGACCTAGAACTGCGGCT
hMRP4	F: TGCAAGGGTTCTGGGATAAAGA
hMRP4	R: CTTTGGCACTTTCCTCAATTAACG
hABCG5	F: CCCAAGGGACTCCGGGGTCA
hABCG5	R: GACCCATGGACCCTCCGGGG
hABCG8	F: CTTCTACCTCGCCGGGGGCT
hABCG8	R: CCGCGATGGTGAGGTTCCCG
hABCA1	F: GAAGTACATCAGAACATGGGC
hABCA1	R: GATCAAAGCCATGGCTGTAG
hABCG1	F: CAGGAAGATTAGACACTGTGG
hABCG1	R: GAAAGGGGAATGGAGAGAAG
hLDLR	F: CCCAGCGAAGATGCGAAGATAT
hLDLR	R: GGTGAAGAAGAGGTAGGCGATG
hABCB4	F: AACCCCAAGATCCTTCTGCT
hABCB4	R: GGACCGTAGACAGTCGGTGT
hCYP27A1	F: AAGCGATACCTGGATGGTTG
hCYP27A1	R: TGTTGGATGTCGTGTCCACT
hGAPDH	F: ACCATCATCCCTGCCTCTAC
hGAPDH	R: CCTGTTGCTGTAGCCAAAT

m: mouse; h: human.

* The oligos for PPARγ and FXR were for the total amount (all isoforms) since they are designed to measure the conserved regions of the genes.
